# Propensity score analysis of red cell distribution width to serum calcium ratio in acute myocardial infarction as a predictor of in-hospital mortality

**DOI:** 10.3389/fcvm.2023.1292153

**Published:** 2023-12-19

**Authors:** Sulan Huang, Huijia Zhang, Zhijie Zhuang, Ning Guo, Quan Zhou, Xiangjie Duan, Liangqing Ge

**Affiliations:** ^1^ The First Affiliated Hospital, Jinan University, Guangzhou, China; ^2^ Department of Cardiovascular Medicine, The First People's Hospital of Changde City, Changde City, Hunan Province, China; ^3^ Department of Rheumatology and Immunology, The First People's Hospital of Changde City, Changde City, Hunan Province, China; ^4^ Department of Gastroenterology, The First People's Hospital of Changde City, Changde City, Hunan Province, China; ^5^ Department of Science and Education Section, The First People's Hospital of Changde City, Changde City, Hunan Province, China; ^6^ Department of Infectious Disease, The First People's Hospital of Changde, Changde City, Hunan Province, China

**Keywords:** red cell distribution width, serum calcium, in-hospital mortality, acute myocardial infarction, propensity score analysis

## Abstract

**Objective:**

Red cell distribution width (RDW) and serum calcium (Ca) levels are predictors of in-hospital mortality in acute myocardial infarction (AMI) patients. However, their sensitivity and specificity are limited. Therefore, this study aimed to determine whether the RDW to Ca ratio (RCR) acquired on admission can be used to predict the in-hospital mortality of AMI patients.

**Methods:**

This retrospective cohort study extracted clinical information from the Medical Information Market for Intensive IV (MIMIC-IV) database on 2,910 AMI patients enrolled via propensity score matching (PSM). Prognostic values were assessed using a multivariate logistic model and three PSM approaches. Analysis was performed based on stratified variables and interactions among sex, age, ethnicity, anemia, renal disease, percutaneous transluminal coronary intervention (PCI), coronary artery bypass grafting (CABG), atrial fibrillation, congestive heart failure, dementia, diabetes, paraplegia, hypertension, cerebrovascular disease, and Sequential Organ Failure Assessment (SOFA) score.

**Results:**

A total of 4,105 ICU-admitted AMI patients were analyzed. The optimal cut-off value of the RCR for in-hospital mortality was 1.685. The PSM was performed to identify 1,455 pairs (2,910) of score-matched patients, with balanced differences exhibited for nearly all variables.The patients’ median age was 72 years (range, 63–82 years) and 60.9% were male. The risk of in-hospital mortality incidence increased with increasing RCR levels. After adjusting for confounders, the risk ratio for the incidence of in-hospital mortality for high RCR was 1.75 [95% confidence interval (CI): 1.60–1.94, *P* = 0.0113] compared to that associated with low RCR in the PSM cohort. High RCR was also substantially implicated in in-hospital mortality incidence in the weighted cohorts [odds ratio (OR) = 1.76, 95% CI: 1.62–1.94, *P* = 0.0129]. Assessment of RCR in three groups showed that patients with high RCR also had a higher risk of in-hospital mortality (OR = 3.04; 95% CI, 2.22–4.16; *P* < 0.0001) than in patients with RCR in the adjusted model. In the sensitivity analysis, both the original and weighted groups showed similar results.

**Conclusion:**

The RCR at admission may be useful for predicting in-hospital mortality in ICU-admitted AMI patients.

## 1. Introduction

Acute myocardial infarction (AMI) is the leading cause of death due to cardiovascular disease (1). An estimated 800,000 people in the United States alone develop AMI annually (2, 3). Despite significant advances in medical technology, particularly in the field of coronary revascularization, the mortality rate of AMI remains concerning (4). Severely ill AMI patients experience a greater occurrence of death and complications owing to the sudden worsening of symptoms and a limited window for providing life-saving interventions (5, 6). Early detection and effective management are essential for preventing life-threatening circumstances and improving patient prognosis. The development of non-invasive, inexpensive tests to identify patients at increased risk of in-hospital mortality is essential to further reduce in-hospital mortality.

Coronary atherosclerosis is a chronic disease which has stable and unstable periods. People during an unstable period may develop a MI if the vascular wall is stimulated by inflammation. Red cell distribution width (RDW) can reflect the inflammatory state of the body, suggesting that it can be used as a marker of inflammation (7). RDW measures the variability in circulating erythrocyte size and is associated with increased in-hospital mortality rates in AMI patients (7–10). Additionally, Cao et al. found that high serum calcium (Ca) levels are also associated with an increased risk of death from any cause and cardiac death after AMI (11). Shiyovich et al. also suggested that serum Ca level is an independent predictor of in-hospital mortality after AMI, showing a U-shaped relationship (12). Many prospective studies have found an association between serum calcium and in-hospital mortality in patients with AMI (12). Although RDW and Ca levels can serve as indicators of AMI severity, their sensitivity and specificity are relatively low (13, 14). The RDW/Ca ratio(RCR) is defined as the ratio of RDW to serum Ca. The RCR is a widely used and straightforward index in clinical settings as it solely necessitates venous blood. As a new biomarker, the RCR has emerged as an independent prognostic indicator of poor outcomes in acute pancreatitis (13). An elevated RCR can quickly, conveniently, economically, sensitively, and accurately predict severity and mortality in patients with acute pancreatitis (13, 14).

To date, no study has evaluated the association between RCR and in-hospital mortality among AMI patients. Thus, the present study aimed to examine the prognostic significance of RCR in a large population of ICU-admitted AMI patients and identified high-risk patients using a simple and convenient indicator.

## 2. Materials and methods

### 2.1. Study design

Data were gathered from the Medical Information Mart for Intensive Care IV (MIMIC-IV) database, a retrospective cohort study that included critical care information of 76,540 patients who had been admitted to intensive care units and treated in hospitals at the Beth Israel Deaconess Medical Center (Boston, MA, USA) from 2008 to 2019 (15). One author (XD) had access to the data and was responsible for its extraction (certification number: 6182750).

### 2.2. Study population

**Figure 1:**
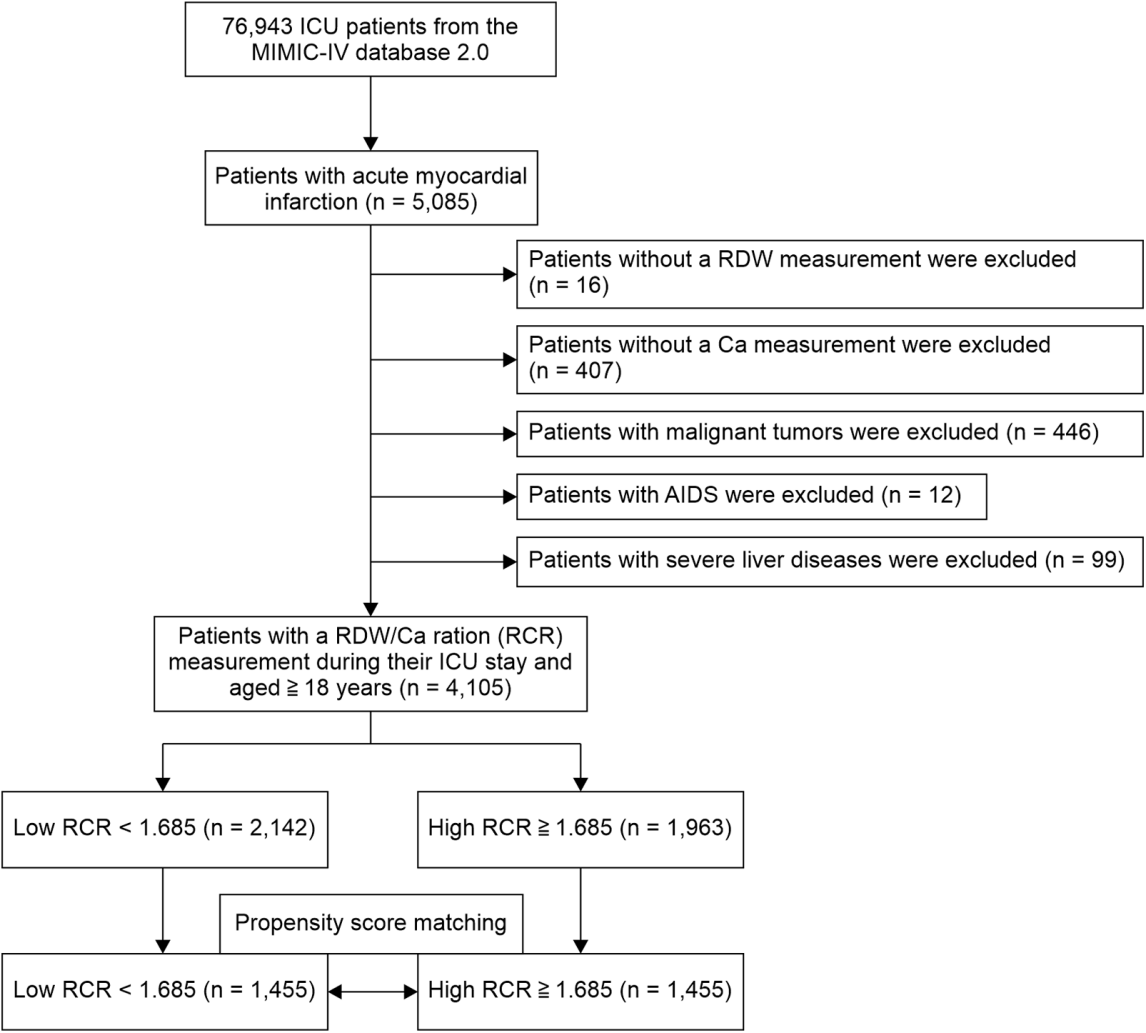
Participant flowchart. The analysis included a total of 4,105 patients with acute myocardial infarction (AMI).

The participants underwent at least one RDW and Ca measurement during the admission period. We observed the participants until they died or were discharged. We included adult patients aged ≥18 years with AMI based on International Classification of Diseases, 9th revision (ICD-9) codes 410.0 (410.00, 410.01, and 410.02), 410.1 (410.10, 410.11, and 410.12), 410.2 (410.20, 410.21, and 410.22), 410.3 (410.30, 410.31, and 410.32), 410.4 (410.40, 410.41, and 410.42), 410.5 (410.50, 410.51, and 410.52), 410.6 (410.60, 410.61, and 410.62), 410.7 (410.70, 410.71, and 410.72), 410.8 (410.80, 410.81, and 410.82), and 410.9 (410.90, 410.91, and 410.92), and ICD-10 codes: I21.0 (I21.01, I21.02, and I21.09), I21.1 (I21.11 and I21.19), I21.2 (I21.21 and I21.29), I21.3, and I21.4. We identified 5,085 ICU-admitted AMI patients. 1,458 patients came from coronary care unit(CCU). The exclusion criteria included patients (1) admitted to the hospital without an RDW measurement (*n* = 16), (2) who did not undergo Ca measurement during their hospital stay (*n* = 407), (3) with malignant tumors (*n* = 446), (4) with AIDS (*n* = 12), and (5) with severe liver diseases (*n* = 99). This study included 4,105 AMI patients (Figure 1). As a baseline, data from the first 24 h after ICU admission were collected. Troponin T were peak levels.

### 2.3. AMI definition

AMI was defined as an increase or decrease in troponin T values with at least one value exceeding the 99th percentile upper reference limit and at least one of the following symptoms of myocardial ischemia: development of pathological Q-wave imaging evidence of new loss of viable myocardium, new ischemic electrocardiographic changes, or an abnormality of regional wall motion consistent with an ischemic cause (16).

### 2.4. Clinical endpoint measurements

In-hospital death was defined as death due to any cause during admission.To make the data more reliable, serum calcium (mg/ml) was corrected to albumin (g/dl) using the Payne formula: corrected serum calcium (corrected sCa) = Serum calcium + 0.8 × (4.0—albumin) (12).

### 2.5. Statistical analyses

Variables with continuous values were reported as averages ± standard deviations (SD) or medians (interquartile range). Based on the normality of the data distribution, Student's *t*- or Mann–Whitney *U*-tests were used for comparisons. Categorical variables were analyzed using the chi-square test and reported as case numbers (%).

By calculating the maximum Youden index for survival status, the optimal cutoff value of RCR was determined using receiver operating curve (ROC) curve analysis, in which the Youden index was equal to the sum of sensitivity and specificity minus 1. The best cutoff value of RCR was 1.685, with 88.1% sensitivity, 37.1% specificity, 28.7% positive predictive value, 88.2% negative predictive value, and 0.252 Youden index. The patients were divided into two groups according to the RCR cutoff value.

Propensity score matching (PSM) was employed because it was difficult to achieve complete stochasticity during patient screening. PSM balances the selection bias and underlying confounding factors. PSM analysis was conducted in the present study using a logistic regression model that included patient age and sex, the presence of congestive heart failure, mild liver disease, renal disease, peripheral vascular disease, cerebrovascular disease, dementia, chronic pulmonary disease, rheumatic disease, peptic ulcer disease, and diabetes, mean corpuscular volume (MCV), mean corpuscular-hemoglobin concentration (MCHC), Sequential Organ Failure Assessment (SOFA) score, thiazide use, and parathyroid diseases. In order to assess PSM levels, we used standardized mean differences (SMDs). For a given covariate, an SMD *<*10.0% represents a relatively small imbalance (17, 18). We considered a threshold of 0.01 to be an acceptable PSM degree. Matching was conducted with a caliper of 0.01 for patients grouped into low RCR (<1.685) and high RCR (≥1.685) pairs. A total of 2,910 propensity score-matched patients (1,455 pairs) with matching propensity scores were identified. Univariate and multivariate logistic regression analyses were performed to evaluate the prognostic relevance of the RCR. After considering clinical significance, the final adjusted model retained the covariates that caused significant changes in the effect estimate of >10% (*P* < 0.05) (19). The propensity score (PS) was estimated to perform sensitivity analyses to calculate the inverse probability of treatment weight (IPTW). In this example, 1/PS is the weight of high RCR, whereas 1/(1–PS) corresponds to the weight of low RCR. The IPTW model was then used to create a weighted cohort. Sensitivity analysis was conducted using two relationship inference models in the original and weighted cohorts, respectively (20, 21). Next, we analyzed the modifications and interactions of the subgroups based on the likelihood ratio test.

The statistical analyses were performed using IBM SPSS Statistics for Windows, version 26.0 (IBM Corp., Armonk, NY, USA), R (http://www.R-project.org, The R Foundation), and Empower Stats software (www.empowerstats.com, X&Y Solutions, Inc., Boston, MA, USA).

## 3. Results

### 3.1. Study population and clinical characteristics

As shown Table 1, the average age of the study population in the original cohort was 71.4 ± 13.3 years. Our preliminary analysis of the confounding variables identified significant differences between the high and low RCR groups before PSM. Patients with high RCR had a lower male ratio, proportion of white ethnicity, systolic blood pressure (SBP), diastolic blood pressure (DBP), Ca, RBC, glucose, and length of hospital stay, had a higher age, male ratio, proportion of white, heart rate, respiratory rate, SOFA, RCR, WBC, creatinine, creatine kinase-myocardial band (CK-MB), and troponin T, and were more likely to have a high prevalence of congestive heart failure, dementia, chronic pulmonary disease, chronic pulmonary disease, mild liver disease, hypertension,cardiogenic shock, and in-hospital mortality (*P* < 0.05 for all).

**Table 1:** Baseline clinical characteristics before and after propensity score matching.

Characteristic	Before PSM				After PSM			
	Low RCR (*n* = 2,142)	High RCR (*n* = 1,963)	SMD	*P* values	Low RCR (*n* = 1,455)	High RCR (*n* = 1,455)	SMD	*P* values
Demographics								
Age, years	71.00 (61.00–80.00)	74.00 (65.00–83.00)	0.24	<0.001	73.00 (64.00–82.00)	73.00 (64.00–82.00)	0.01	0.986
Sex, *n* (%)			0.14	<0.001			0.01	0.851
Male	1,373 (64.10%)	1,127 (57.41%)			855 (58.76%)	860 (59.11%)		
Female	769 (35.90%)	836 (42.59%)			600 (41.24%)	595 (40.89%)		
Ethnicity, *n* (%)			0.10	0.006			0.13	0.002
White	1,377 (64.29%)	1,247 (63.53%)			956 (65.70%)	935 (64.26%)		
Black	330 (15.41%)	368 (18.75%)			217 (14.91%)	281 (19.31%)		
Other	435 (20.31%)	348 (17.73%)			282 (19.38%)	239 (16.43%)		
Smoking			0.03	0.354			0.02	0.557
No	1,404 (65.67%)	1,260 (64.29%)			929 (65.1%)	913 (63.9%)		
Yes	734 (34.33%)	700 (35.71%)			499 (34.9%)	515 (36.1%)		
Vital signs								
Heart rate, beats/min	82.00 (73.00–95.00)	88.36 (19.61) 86.00 (75.00–100.00)	0.18	<0.001	83.00 (74.00–97.00)	85.00 (74.00–99.00)	0.08	0.046
SBP, mmHg	122.00 (107.00–137.00)	116.00 (103.00–134.00)	0.17	<0.001	121.00 (106.00–137.00)	118.00 (104.00–135.00)	0.08	0.009
DBP, mmHg	68.00 (58.00–80.00)	64.00 (54.00–77.00)	0.18	<0.001	66.00 (56.00–79.00)	65.00 (54.00–77.25)	0.08	0.011
Temperature, °C	36.67 (36.39–36.94)	36.67 (36.33–37.00)	0.01	0.152	36.61 (36.39–36.94)	36.67 (36.39–37.00)	0.05	0.036
SPO_2_, %	98.00 (95.00–100.00)	98.00 (95.00–100.00)	0.11	0.070	98.00 (95.00–100.00)	98.00 (95.00–100.00)	0.06	0.219
Respiratory rate, breaths/min	18.00 (15.00–22.00)	19.00 (16.00–23.00)	0.18	<0.001	18.00 (15.00–23.00)	19.00 (16.00–23.00)	0.08	0.015
Scores								
SOFA	4.00 (2.00–7.00)	6.00 (4.00–9.00)	0.54	<0.001	5.00 (3.00–8.00)	5.00 (3.00–8.00)	0.02	0.251
Laboratory results								
RCR	1.53 (1.45–1.61)	1.89 (1.77–2.09)	1.96	<0.001	1.75 (1.60–1.92)	1.60 (1.49–1.81)	0.03	<0.001
WBC, 10^9^/L	11.20 (8.50–14.78)	11.80 (8.50–16.40)	0.17	0.002	11.40 (8.60–15.20)	11.70 (8.30–15.80)	0.07	0.634
RBC, 10^12^/L	4.03 (3.44–4.55)	3.45 (2.92–4.05)	0.60	<0.001	3.91 (3.36–4.44)	3.48 (2.92–4.06)	0.04	<0.001
Creatinine, umol/L	1.10 (0.80–1.50)	1.40 (1.00–2.40)	0.39	<0.001	1.10 (0.90–1.70)	1.30 (0.90–2.10)	0.04	<0.001
Potassium, mmol/L	4.20 (3.90–4.70)	4.30 (3.90–4.90)	0.13	<0.001	4.30 (3.90–4.70)	4.30 (3.90–4.80)	0.01	0.898
Glucose, mg/dl	144.00 (115.25–194.75)	142.00 (110.00–196.00)	0.06	0.019	148.00 (117.00–206.00)	140.00 (109.00–190.00)	0.14	<0.001
ALT, U/L	31.00 (20.00–62.75)	31.00 (17.00–79.00)	0.14	0.984	34.00 (18.00–80.00)	30.00 (19.00–62.00)	0.11	0.055
AST, U/L	55.00 (31.00–143.00)	57.00 (30.00–143.00)	0.10	0.960	61.00 (33.00–160.00)	53.00 (30.00–125.50)	0.09	0.004
CK-MB, mmol/L	0.49 (0.01–51.84)	0.49 (0.01–51.84)	0.28	<0.001	13.00 (5.00–46.00)	12.00 (5.00–41.00)	<0.01	0.730
Troponin T,	0.35 (0.12–1.25)	0.63 (0.18–2.10)	0.20	<0.001	0.43 (0.14–1.59)	0.50 (0.16–1.60)	0.04	0.223
Comorbidities, *n* (%)								
Congestive heart failure			0.32	<0.001			0.04	0.290
No	1,103 (51.49%)	701 (35.71%)			604 (41.51%)	576 (39.59%)		
Yes	1,039 (48.51%)	1,262 (64.29%)			851 (58.49%)	879 (60.41%)		
Peripheral vascular disease			0.19	<0.001			<0.01	0.922
No	1,858 (86.74%)	1,565 (79.72%)			1,205 (82.82%)	1,207 (82.96%)		
Yes	284 (13.26%)	398 (20.28%)			250 (17.18%)	248 (17.04%)		
Dementia			0.08	0.010			0.03	0.380
No	2,062 (96.27%)	1,857 (94.60%)			1,382 (94.98%)	1,392 (95.67%)		
Yes	80 (3.73%)	106 (5.40%)			73 (5.02%)	63 (4.33%)		
Cerebrovascular disease			0.04	0.178			0.03	0.416
No	1,837 (85.76%)	1,654 (84.26%)			1,217 (83.64%)	1,233 (84.74%)		
Yes	305 (14.24%)	309 (15.74%)			238 (16.36%)	222 (15.26%)		
Chronic pulmonary disease			0.27	<0.001			0.04	0.251
No	1,676 (78.24%)	1,302 (66.33%)			1,051 (72.23%)	1,023 (70.31%)		
Yes	466 (21.76%)	661 (33.67%)			404 (27.77%)	432 (29.69%)		
Peptic ulcer disease			0.19	<0.001			0.07	0.064
No	2,116 (98.79%)	1,877 (95.62%)			1,429 (98.21%)	1,414 (97.18%)		
Yes	26 (1.21%)	86 (4.38%)			26 (1.79%)	41 (2.82%)		
Mild liver disease			0.24	<0.001			0.02	<0.001
No	2,074 (96.83%)	1,792 (91.29%)			1,403 (96.43%)	1,336 (91.82%)		
Yes	68 (3.17%)	171 (8.71%)			52 (3.57%)	119 (8.18%)		
Hypertension			0.30	<0.001			0.04	0.254
No	1,343 (68.42%)	1,154 (53.87%)			904 (62.13%)	874 (60.07%)		
Yes	620 (31.58%)	988 (46.13%)			551 (37.87%)	581 (39.93%)		
Diabetes			0.03	0.381			0.01	0.737
No	1,225 (57.19%)	1,096 (55.83%)			793 (54.50%)	802 (55.12%)		
Yes	917 (42.81%)	867 (44.17%)			662 (45.50%)	653 (44.88%)		
Hyperlipidemia			0.19	<0.0001			0.14	<0.001
No	788 (36.79%)	902 (45.95%)			552 (37.94%)	655 (45.02%)		
Yes	1,354 (63.21%)	1,061 (54.05%)			903 (62.06%)	800 (54.98%)		
Hyperparathyroidism			0.04	0.207			0.01	0.080
No	1,829 (85.55%)	1,649 (84.13%)			1,234 (84.99%)	1,231 (84.66%)		
Yes	309 (14.45%)	311 (15.87%)			218 (15.01%)	233 (15.34%)		
Cardiogenic shock			0.13	<0.001			0.02	0.550
No	2,030 (94.77%)	1,797 (91.59%)			92 (6.32%)	100 (6.87%)		
Yes	112 (5.23%)	165 (8.41%)			1,363 (93.68%)	1,335 (93.13%)		
Clinical interventions, *n* (%)								
PTCA			0.02	0.092			0.12	<0.001
No	2,110 (98.51%)	1,945 (99.08%)			1,445 (99.31%)	1,424 (97.87%)		
Yes	32 (1.49%)	18 (0.92%)			10 (0.69%)	31 (2.13%)		
PCI			0.01	0.803			0.03	0.496
No	1,690 (78.90%)	1,555 (79.22%)			1,139 (78.28%)	1,154 (79.31%)		
Yes	452 (21.10%)	408 (20.78%)			316 (21.72%)	301 (20.69%)		
GABG			0.03	0.292			0.01	0.864
No	1,614 (75.35%)	1,451 (73.92%)			1,091 (74.98%)	1,087 (74.71%)		
Yes	528 (24.65%)	512 (26.08%)			364 (25.02%)	368 (25.29%)		
Diuretic			0.04	0.180			0.02	0.583
No	2,112 (98.60%)	1,925 (98.06%)			1,430 (98.28%)	1,426 (98.01%)		
Yes	30 (1.40%)	38 (1.94%)			25 (1.72%)	29 (1.99%)		
In-hospital mortality, *n* (%)			0.41	<0.001			0.12	<0.001
No	1,885 (88.00%)	1,415 (72.08%)			1,130 (77.66%)	1,201 (82.54%)		
Yes	257 (12.00%)	548 (27.92%)			325 (22.34%)	254 (17.46%)		
Length of hospital stay, days	7.13 (4.26,13.19)	6.23 (4.10, 11.68)	0.02	<0.001	7.22 (4.26, 13.32)	6.41 (4.17, 11.74)	0.03	0.016
AMI group, *n* (%)			0.05	0.341			0.04	0.490
STEMI	1,220 (56.96%)	1,075 (54.76%)			799 (54.91%)	829 (56.98%)		
Non-STEMI	670 (31.28%)	638 (32.50%)			470 (32.30%)	455 (31.27%)		
Type 2 AMI	252 (11.76%)	250 (12.74%)			186 (12.78%)	171 (11.75%)		

Data are expressed as median (Q1–Q3) or numbers (%). RCR, red cell distribution width to serum Ca ratio; PSM, propensity score matching; SBP, systolic blood pressure; DBP, diastolic blood pressure; SPO_2_, blood oxygen saturation; SOFA, Sequential Organ Failure Assessment; RCR, red blood distribution width to serum calcium; WBC, white blood cell; RBC, red blood cell; RDW, red blood cell distribution width; ALT, alanine transaminase; AST, aspartate transaminase; CK-MB, creatine kinase-MB; PTCA, percutaneous transluminal coronary angioplasty; PCI, percutaneous transluminal coronary intervention; CABG, coronary artery bypass grafting; AMI, acute myocardial infarction; SMD, standardized mean difference.

The PSM-matched population showed differences in the proportions of white ethnicity, heart rate, SBP, DBP, temperature, respiratory rate, RCR, RBC, creatinine level, glucose level, aspartate transaminase (AST) level, percutaneous transluminal coronary angioplasty (PTCA), prevalence of mild liver disease, hyperlipidemia, in-hospital mortality, and length of hospital stay (all *P* < 0.05).

### 3.2. Association between RCR and in-hospital mortality in AMI patients in the Ps-matched cohort

Table 2 shows the estimated odds ratios (ORs) and 95% confidence interval (CIs) for RCR in relation to in-hospital mortality after PSM. As a categorical variable, the prevalence of in-hospital mortality increased remarkably among patients in the high RCR group compared to that observed in the low RCR group (crude model: OR = 1.74, 95% CI: 1.61–1.88, *P* = 0.0010; Model I: OR = 1.74, 95% CI: 1.61–1.89, *P* = 0.0013; Model II: OR = 1.75, 95% CI: 1.60–1.94, *P* = 0.0113). As a successive variable, after adjusting for the clinical confounders listed, an SD increase in serum uric acid (SUA) level was related to a 158% increased risk of in-hospital mortality (OR = 2.58; 95% CI:1.92–3.47; *P* < 0.0001) in the fully adjusted model. When RCR was assessed in the three models, patients with high RCR also had a higher risk of in-hospital mortality (OR = 3.04; 95% CI, 2.22–4.16; *P* < 0.0001) than that observed in patients in the low RCR group in the adjusted model.

**Table 2:** Adjusted odds ratios for the incidence of in-hospital mortality according to the presence of high RCR in the PS-matched cohort.

Exposure	Non-adjusted		Model I		Model II	
	OR (95% CI)	*P*-value	OR (95% CI)	*P*-value	OR (95% CI)	*P*-value
RCR group						
Low	1.0		1.0		1.0	
High	1.74 (1.61–1.88)	0.0010	1.74 (1.61–1.89)	0.0013	1.75 (1.60–1.94)	0.0113
RCR as continuous variable (mmol/L)	3.85 (2.95–5.01)	<0.0001	3.56 (2.72–4.66)	<0.0001	2.58 (1.92­–3.47)	<0.0001
RCR per SD increase	1.56 (1.43–1.70)	<0.0001	1.52 (1.39–1.66)	<0.0001	1.36 (1.24–1.50)	<0.0001
RCR tertile						
1	1.0		1.0		1.0	
2	1.72 (1.33–2.25)	<0.0001	1.65 (1.26–2.15)	0.0002	1.66 (1.20–2.29)	0.0021
3	3.70 (2.99–4.73)	<0.0001	3.45 (2.67–4.46)	<0.0001	3.04 (2.22–4.16)	<0.0001
*P* for trend	1.95 (1.73–2.20)	<0.0001	1.89 (1.67–2.15)	<0.0001	1.76 (1.51–2.05)	<0.0001

Models were derived from logistic regression models; the non-adjusted model was adjusted for no variable. Model I was adjusted for age, sex, and ethnicity. Model II was adjusted for age, sex, ethnicity, smoking, SOFA, albumin, creatinine, potassium, AST/ALT, hypertension, diabetes, hyperlipidemia, AMI group, PCI, and GABG. RCR, red cell distribution width to serum Ca ratio; OR, odds ratio, CI, confidence interval, SD, standard deviation; SOFA, Sequential Organ Failure Assessment; AST, aspartate aminotransferase; ALT, alanine aminotransferase; PCI, percutaneous transluminal coronary intervention; CABG, coronary artery bypass grafting.

### 3.3. Sensitivity analysis

According to these findings, a sensitivity analysis was applied to further confirm the association between RCR and the incidence of in-hospital mortality in the two cohorts examined, and a weighted cohort was developed with the estimated PS through the development of an IPTW model (Table 3). We also used non-adjusted, partially adjusted, and fully adjusted models across the two cohorts. In the original cohort, a high risk of in-hospital mortality was noted in cases with a high RCR (crude model: OR = 1.73, 95% CI:1.63–1.86, *P* < 0.001; Model I: OR = 1.75, 95% CI:1.64–1.88, *P* < 0.001; Model II: OR = 1.74, 95% CI: 0.54–2.02, *P* = 0.0635) after adjusting for different covariates. The results remained marked in the weighted cohort (crude model: OR = 1.78, 95% CI:1.70–1.87, *P* < 0.001; Model I: OR = 1.78, 95% CI: 1.72–1.88, *P* < 0.001; Model II: OR = 1.76, 95% CI: 1.62–1.94, *P* = 0.0129). The results were also significant when SUA was used as a successive variable. When RCR was assessed in the three models, patients with high RCR also had a higher risk of in-hospital mortality than that observed in patients with low RCR in both original and weighted cohorts.

**Table 3:** Association of RCR and in-hospital mortality among AMI in the original (A) and weighted cohorts (B).

Exposure (A)	Non-adjusted		Model I		Model II	
	OR (95% CI)	*P*-value	OR (95% CI)	*P*-value	OR (95% CI)	*P*-value
RCR group						
Low	1.0		1.0		1.0	
High	1.73 (1.63–1.86)	<0.0001	1.75 (1.64–1.88)	<0.0001	1.74 (0.54–2.02)	0.0635
RCR as continuous variable (mmol/L)	3.64 (2.91–4.55)	<0.0001	3.36 (2.68–4.22)	<0.0001	1.98 (1.36–2.90)	0.0004
RCR per SD increase	1.52 (1.42–1.64)	<0.0001	1.48 (1.38–1.60)	<0.0001	1.25 (1.10–1.41)	0.0004
RCR tertile						
1	1.0		1.0		1.0	
2	1.57 (1.26–1.95)	<0.0001	1.50 (1.20–1.88)	0.0004	1.43 (0.95–2.14)	0.0840
3	3.4 (2.77–4.16)	<0.0001	3.17 (2.56–3.93)	<0.0001	2.71 (1.82–4.03)	<0.0001
*P* for trend	1.88 (1.70–2.08)	<0.0001	1.82 (1.64–2.03)	<0.0001	1.67 (1.37–2.04)	<0.0001
Exposure (B)	Non-adjusted		Model I		Model II	
	OR (95% CI)	*P*-value	OR (95% CI)	*P*-value	OR (95% CI)	*P*-value
RCR group						
Low	1.0		1.0		1.0	
High	1.78 (1.70–1.87)	<0.0001	1.78 (1.72–1.88)	<0.0001	1.76 (1.62–1.94)	0.0129
RCR as continuous variable (mmol/L)	3.69 (3.17–4.29)	<0.0001	3.44 (2.95–4.02)	<0.0001	1.98 (1.53–2.56)	<0.0001
RCR per SD increase	1.53 (1.45–1.62)	<0.0001	1.49 (1.42–1.57)	<0.0001	1.25 (1.15–1.36)	<0.0001
RCR tertile						
1	1.0		1.0		1.0	
2	1.59 (1.35–1.86)	<0.0001	1.52 (1.29–1.78)	<0.0001	1.48 (1.11–1.96)	0.0074
3	3.59 (3.11–4.15)	<0.0001	3.36 (2.89–3.91)	<0.0001	2.73 (2.07–3.60)	<0.0001
*P* for trend	1.95 (1.81–2.09)	<0.0001	1.89 (1.75–2.03)	<0.0001	1.67 (1.46–1.92)	<0.0001

Models were derived from logistic regression models; the non-adjusted model was adjusted for none. Adjust I model adjusted for age, sex, and ethnicity; Adjust II model adjusted for age, sex, ethnicity, smoking, SOFA, albumin, creatinine, potassium, AST/ALT, hypertension, diabetes, hyperlipidemia, AMI group, PCI, GABG,RCR, red cell distribution width to serum Ca ratio; OR, odds ratio, CI, confidence interval; SD, standard deviation; SOFA, Sequential Organ Failure Assessment; AST, aspartate aminotransferase; ALT, alanine aminotransferase; PCI, percutaneous transluminal coronary intervention; CABG, coronary artery bypass grafting.

### 3.4. E-value analyses

**Figure 2:**
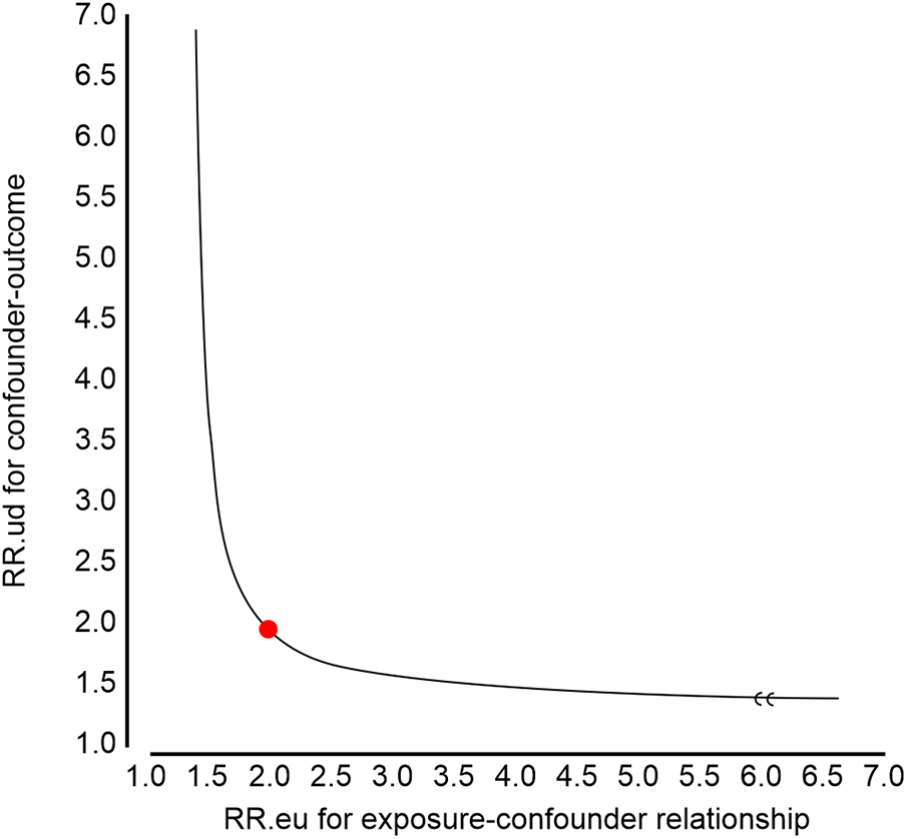
E-value analyses. An unmeasured confounder could fully account for the association of intensification with red cell distribution width to serum calcium ratio (RCR) if it was associated with the exposure and outcome with an odds ratio (OR) of 1.98 (lower confidence limit, 1.59).

Using Van der Weele and Ding's E-value methodology, we conducted a sensitivity analysis to identify possible confounding effects (22). In the current study, the adjusted OR for the associations between RCR and in-hospital mortality was 1.75 (95% CI 1.60–1.94). An unmeasured confounder could fully account for the association of intensification with in-hospital mortality if it was associated with the exposure and outcome with an OR of 1.98 (lower confidence limit, 1.59) (Figure 2). Considering this E-value, fewer confounding factors seemed to affect the current association between RCR and in-hospital mortality.

### 3.5. Subgroup analysis

**Figure 3:**
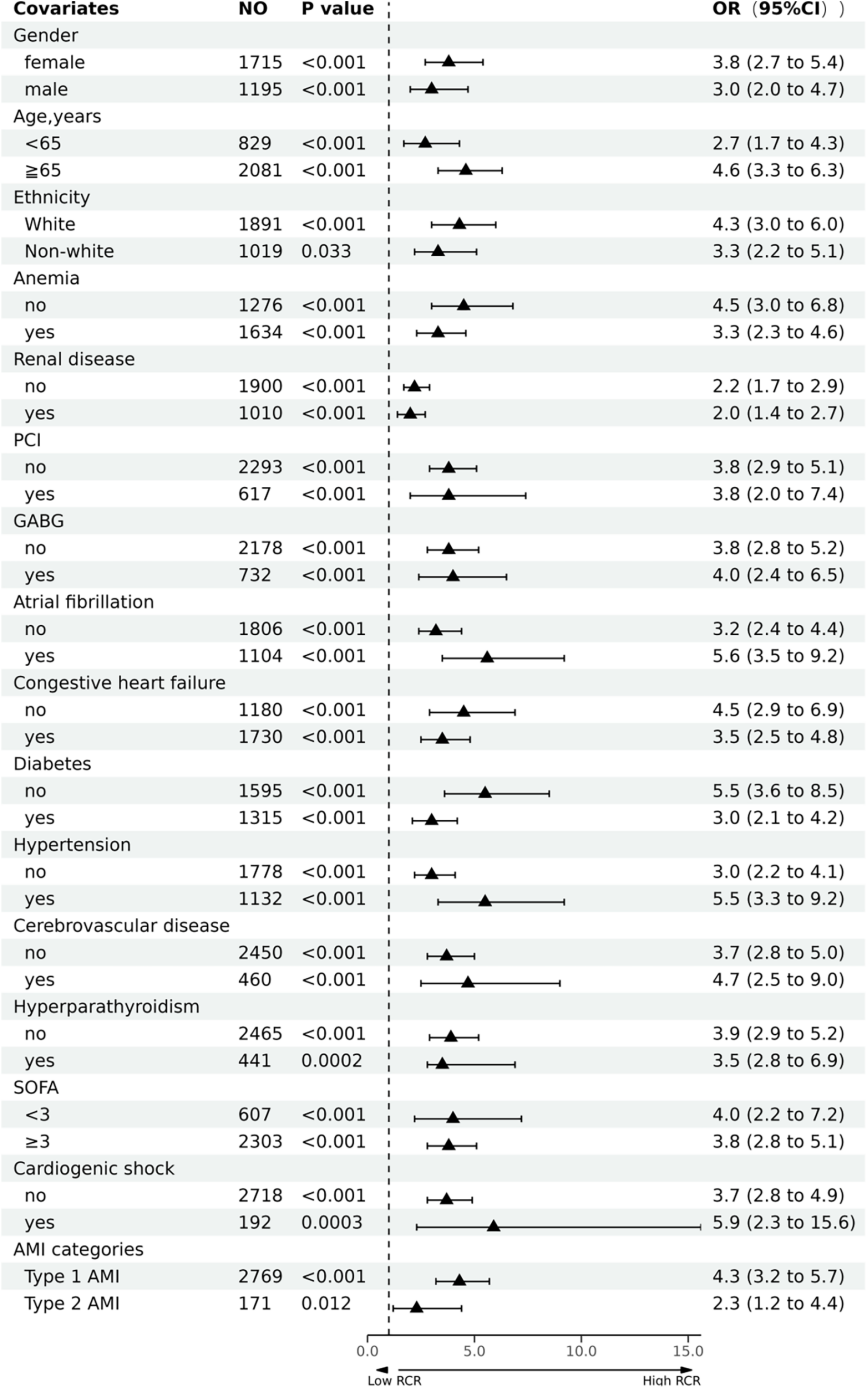
Forest plot of the association between red cell distribution width to serum calcium ratio (RCR) and in-hospital mortality of patients with acute myocardial infarction (AMI).

We performed stratified analysis to evaluate the robustness of our findings in diverse subgroups following PSM. After adjusting for potential confounders, we found no interactions between sex, age, ethnicity, anemia, renal disease, PCI, CABG, atrial fibrillation, congestive heart failure, diabetes, hypertension, cerebrovascular disease, hyperparathyroidism, SOFA score, cardiogenic shock, and AMI categories (Table 4 and Figure 3). The high RCR (≥1.685) group had a higher in-hospital mortality rate than that in the low RCR (<1.685) group for all subgroups. However, whether RCR interacted with any of the subgroup factors (*P* > 0.05) was not evident when we analyzed the interaction between RCR and each factor.

**Table 4:** Stratified analysis for treatment effect for each covariate.

Covariates	No	OR (95% CI)	Subgroup *P*-value	*P* for interaction
Sex				0.3859
Female	1,715	3.8 (2.7–5.4)	<0.001	
Male	1,195	3.0 (2.0–4.7)	<0.001	
Age, years				0.0700
<65	829	2.7 (1.7–4.3)	<0.001	
≧65	2,081	4.6 (3.3–6.3)	<0.001	
Ethnicity				0.3685
White	1,891	4.3 (3.0–6.0)	<0.001	
Non-white	1,019	3.3 2.2–5.1)	0.033	
Anemia				0.2366
No	1,276	4.5 (3.0, 6.8)	<0.001	
Yes	1,634	3.3 (2.3, 4.6)	<0.001	
Renal disease				0.5658
No	1,900	2.2 (1.7–2.9)	<0.001	
Yes	1,010	2.0 (1.4–2.7)	<0.001	
PCI				0.9987
No	2,293	3.8 (2.9–5.1)	<0.001	
Yes	617	3.8 (2.0–7.4)	<0.001	
GABG				0.8793
No	2,178	3.8 (2.8–5.2)	<0.001	
Yes	732	4.0 (2.4–6.5)	<0.001	
Atrial fibrillation				0.0590
No	1,806	3.2 (2.4–4.4)	<0.001	
Yes	1,104	5.6 (3.5–9.2)	<0.001	
Congestive heart failure				0.3609
No	1,180	4.5 (2.9–6.9)	<0.001	
Yes	1,730	3.5 (2.5–4.8)	<0.001	
Diabetes				0.1372
No	1,595	5.5 (3.6–8.5)	<0.001	
Yes	1,315	3.0 (2.1–4.2)	<0.001	
Hypertension				0.0509
No	1,778	3.0 (2.2–4.1)	<0.001	
Yes	1,132	5.5 (3.3–9.2)	<0.001	
Cerebrovascular disease				0.5067
No	2,450	3.7 (2.8–5.0)	<0.001	
Yes	460	4.7 (2.5–9.0)	<0.001	
Hyperparathyroidism				0.7823
No	2,465	3.9 (2.9–5.2)	<0.001	
Yes	441	3.5 (1.8–6.9)	0.0002	
SOFA score				0.9183
<3	607	4.0 (2.2–7.2)	<0.001	
≥3	2,303	3.8 (2.8–5.1)	<0.001	
Cardiogenic shock				0.3564
No	2,718	3.71 (2.82–4.89)	<0.001	
Yes	192	5.92 (2.25–15.58)	0.0003	
AMI categories				0.0912
Type 1 AMI		4.28 (3.20–5.73)	<0.001	
Type 2 AMI		2.31 (1.20, 4.43)	0.0120	

OR, odds ratio; CI, confidence interval; PCI, percutaneous transluminal coronary intervention; CABG, coronary artery bypass grafting; SOFA, sequential organ failure assessment.

### 3.6. Prediction of in-hospital mortality

Finally, we examined the performance of RCR as a predictor of in-hospital mortality. ROC curves were constructed using the RCR in the original and matched populations (Supplementary Figures S1 and S2, respectively). The results showed AUC values of 0.65 (95% CI: 0.63–0.67) and 0.66 (95% CI: 0.63–0.68), respectively.

Furthermore,the predictive values for in-hospital mortality of troponin T and RCR were compared in patients with AMI. ROC curves generated for RCR and troponin T are plotted in Supplementary Figure S3. The AUC for RCR was 0.66 (range, 0.63-0.67), whereas it was 0.51 (range, 0.48-0.53) for troponin T (*P* < 0.001).

Finally, ROC curves were also used to evaluate the prognostic value of RCR, RDW, and Ca in patients with AMI. The AUCs of RCR, RDW, and Ca for predicting the in-hospital mortality were 0.66, 0.65, and 0.53, respectively (Supplementary Figure S4).

## 4. Discussion

To our knowledge, this is the first study to investigate the prognostic value of RCR on admission in a large population of ICU-admitted AMI patients. Our findings demonstrated that AMI patients with a high RCR were at an increased risk of in-hospital mortality. The multivariate models showed adverse outcomes associated with RCR after adjusting for confounding factors. Thus, RCR may be useful to evaluate the prognosis of AMI patients. Additionally, our analysis of the PSM cohorts revealed an association between RCR and in-hospital mortality, in which the risk of in-hospital mortality increased by 75% after adjusting for PS, and the association persisted even after adjusting for all other confounders. Similarly, the original and weighted cohorts showed similar results after sensitivity analysis. The results obtained by stratifying by the chosen variables in addition to adjusting for PS were also consistent. As a further measure of the association of RCR with in-hospital mortality, we analyzed RCR associations in different subgroups of traditional cardiovascular risk factors.

The RCR is a straightforward index widely used in clinical settings because it only requires venous blood. Thus, RDW and Ca are regularly assessed in AMI patients admitted to the intensive care unit (ICU). RCR was a blend of two parameters that had been demonstrated to possess a superior capability in forecasting the disease's prognosis. The RDW, along with other standard complete blood count parameters, is used to identify diseases of the hematological system (23). Various diseases can be prognosticated by RDW, including cardiovascular and cerebrovascular disorders, respiratory diseases, cancer, and critical illness (24–26). The RDW reflects the inflammation level. Inflammatory reactions play a significant role in AMI occurrence and progression, which may lead to an elevation in RDW (8, 27, 28).We believe that the RDW can be used to predict the prognosis of AMI patients because it reflects the level of inflammation. Similarly, serum Ca level is an independent predictor of in-hospital death in patients with ST elevation myocardial infarction (STEMI) (29). Fang et al. reported that both decreased and increased corrected serum Ca levels in AMI patients are associated with increased in-hospital mortality; moreover, patients with corrected serum Ca levels of 2.35 mmol/L showed the lowest risk of in-hospital death (30). As predictors of in-hospital mortality from AMI, most previous studies included either RDW or Ca; however, we also considered the ratio of RDW to Ca. RCR is a blend of two parameters that have been demonstrated to possess superior capability in predicting disease prognosis. However, few studies have investigated the prognostic value of RCR in acute pancreatitis (13, 14, 31, 32). Han et al. suggested that the RCR may play a valuable role in predicting acute pancreatitis severity (31). Additionally, Gravito-Soares et al. conducted a study of 666 acute pancreatitis patients and determined that RCR may be a new method to identify acute pancreatitis patients requiring transferred to the ICU, with accompanying complications requiring surgical treatment, or at risk for early-stage in-hospital death (32). These outcomes suggest that a high RCR is associated with more severe circumstances than those associated with a low RCR. Our study results suggest that RCR can be used for the early identification of ICU-admitted AMI patients with poor prognoses. These findings are consistent with those reported previously. It is suggested that more attention should be given to the effect of RCR levels on the early prognosis of AMI patients.

Our findings revealed higher in-hospital mortality in AMI patients with high RDW and low serum Ca level. However, the mechanism behind the close correlation between RCR and in-hospital mortality in AMI patients is currently unknown. High RDW is associated with a poor prognosis in AMI, but the mechanisms underlying this association are also unknown. This process is influenced by factors that impair the hematopoietic function of the bone marrow. Most of these risk factors are shared with those associated with worsening coronary artery disease prognoses. Several hypotheses have been proposed, including the potential roles of anemia (33), inflammatory cytokines (34), free cholesterol (35), oxidative stress (36), thrombosis (37), nutritional deficiency (8), microvascular disorders (38), and neurohumoral and adrenergic systems (39). AMI patients with low serum Ca levels have higher in-hospital mortality for several reasons. First, these factors are directly related to the electrophysiological properties of cardiomyocyte membranes in which Ca levels are determined. When Ca levels are low, Ca channels may not close as quickly as they should, leading to prolonged plateaus (40). Furthermore, patients with low serum Ca levels are more likely to experience cardiac arrhythmias and arrests (41). Second, there is a possibility of reversing heart failure and cardiomyopathy when Ca levels are low (42). Third, low Ca levels are associated with multiple cardiovascular risk factors including hypertension and decreased left ventricular systolic function (43). Moreover, evidence suggests that Ca administered to STEMI patients with hypocalcemia may improve their short-term outcomes after AMI; however, further research is needed (29).

Few studies have explored the clinical value of RCR in AMI patients. Identifying the precise mechanisms underlying the strong association between RCR and inpatient death in AMI patients is challenging. We demonstrated that RDW/Ca (AUC = 0.66) had better predictive power in predicting in-hospital mortality of AMI than the single factors: RDW(AUC = 0.65, *P* < 0.05) and Ca (AUC = 0.53, *P* < 0.05). We also clarified that RDW/Ca was a risk factor for clinical deterioration in AMI. However, the AUC of RCR was subtle greater than RDW. For a more robust conclusion and to define a precise cut-off value of RCR, we recommend further multicenter prospective trials with a larger sample size.

Our study had several strengths. To our knowledge, this was the first study to examine the relationship between RCR and mortality in a large cohort of ICU-admitted AMI patients. The significant relationship between RCR and in-hospital death remained after a multiple logistic regression analysis adjusted for several confounding factors, indicating a good level of stability. This study used rigorous statistical adjustments to mitigate the residual confounding factors to which observational studies are prone.

However, our study also had several limitations. First, causal inferences were not possible because of the study design. Although PSM is among the most effective methods for mitigating inherent biases in non-randomized studies, caution is needed when interpreting our findings as residual biases might have remained. Second, the analysis might have introduced measurement errors due to the influence of specific factors on RCR and the exclusion of various RCR measurements. Third, data on the clinical outcome of cardiac death, which is more consequential, was not obtained. Finally, not all patients were from CCU, which is potential limitation affecting generalizability. The data were from the United States, and thus the results may not apply fully to ICUs elsewhere with different practices or resources. As a single-center study, caution should be exercised in interpreting findings from other populations and regions. Therefore, large prospective multicenter studies and follow-ups are needed to confirm these results in the future.

## 5. Conclusions

We demonstrated a significant positive association between RCR and in-hospital mortality in ICU-admitted AMI patients. Further research is necessary to determine whether the RCR predicts in-hospital mortality in AMI patients.

## Data Availability

Publicly available datasets were analyzed in this study. This data can be found here: https://mimic.physionet.org.

## Supplementary material

SUPPLEMENTARY FIGURE S1

ROC curve for the prediction of in-hospital mortality in the original population.

SUPPLEMENTARY FIGURE S2

ROC curve for the prediction of in-hospital mortality in the matched population.

SUPPLEMENTARY FIGURE S3

ROC curves of RCR and troponin T.

SUPPLEMENTARY FIGURE S4

ROC curves of RCR, RDW, and Ca.
