# Supplementary figures and images for: Propensity score analysis of red cell distribution width to serum calcium ratio in acute myocardial infarction as a predictor of in-hospital mortality
Source: Front Cardiovasc Med. 2023 Dec 19;10:1292153. doi: 10.3389/fcvm.2023.1292153 (PMC10758436; doi:10.3389/fcvm.2023.1292153)

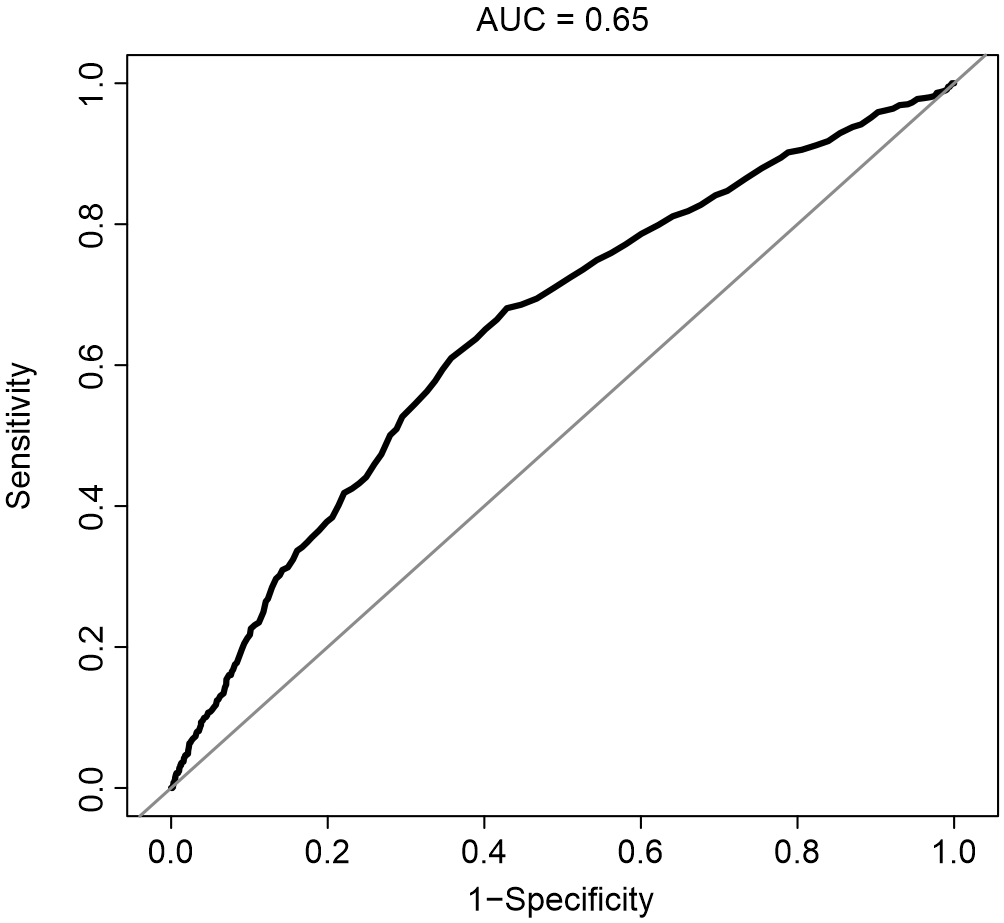

Supplement: Supplementary file 1 [file Image1.tif]

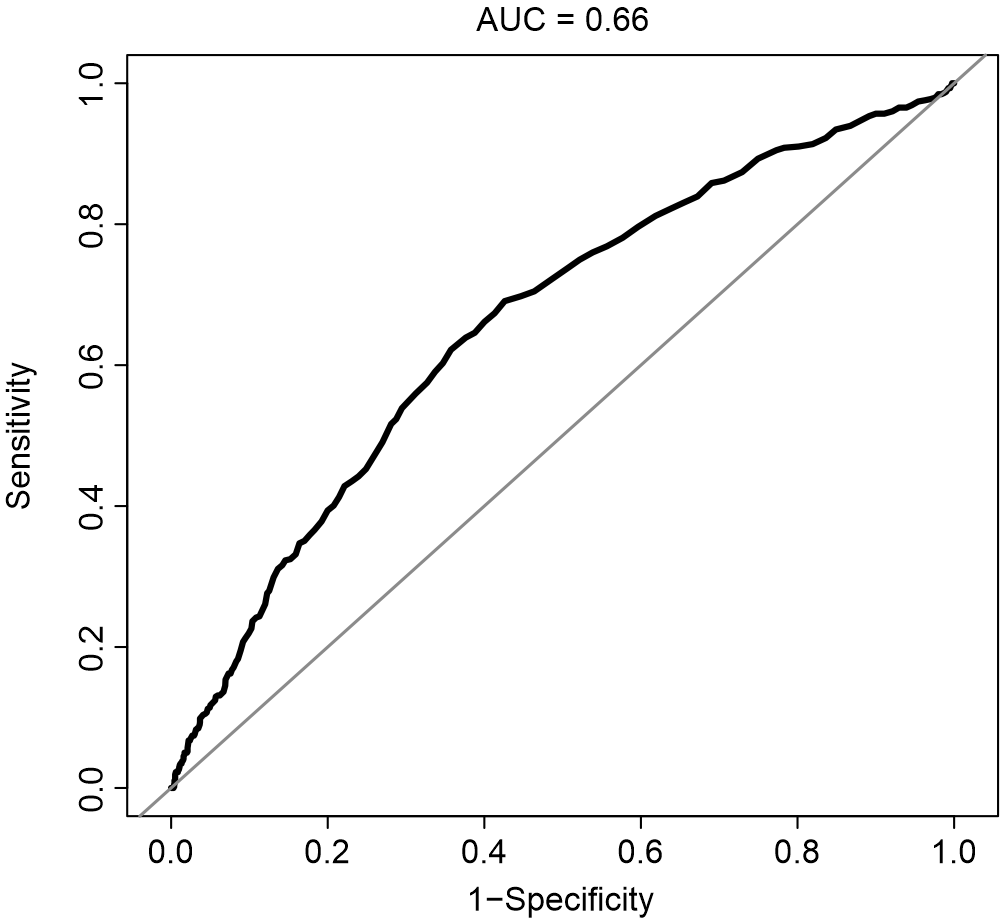

Supplement: Supplementary file 2 [file Image2.tif]

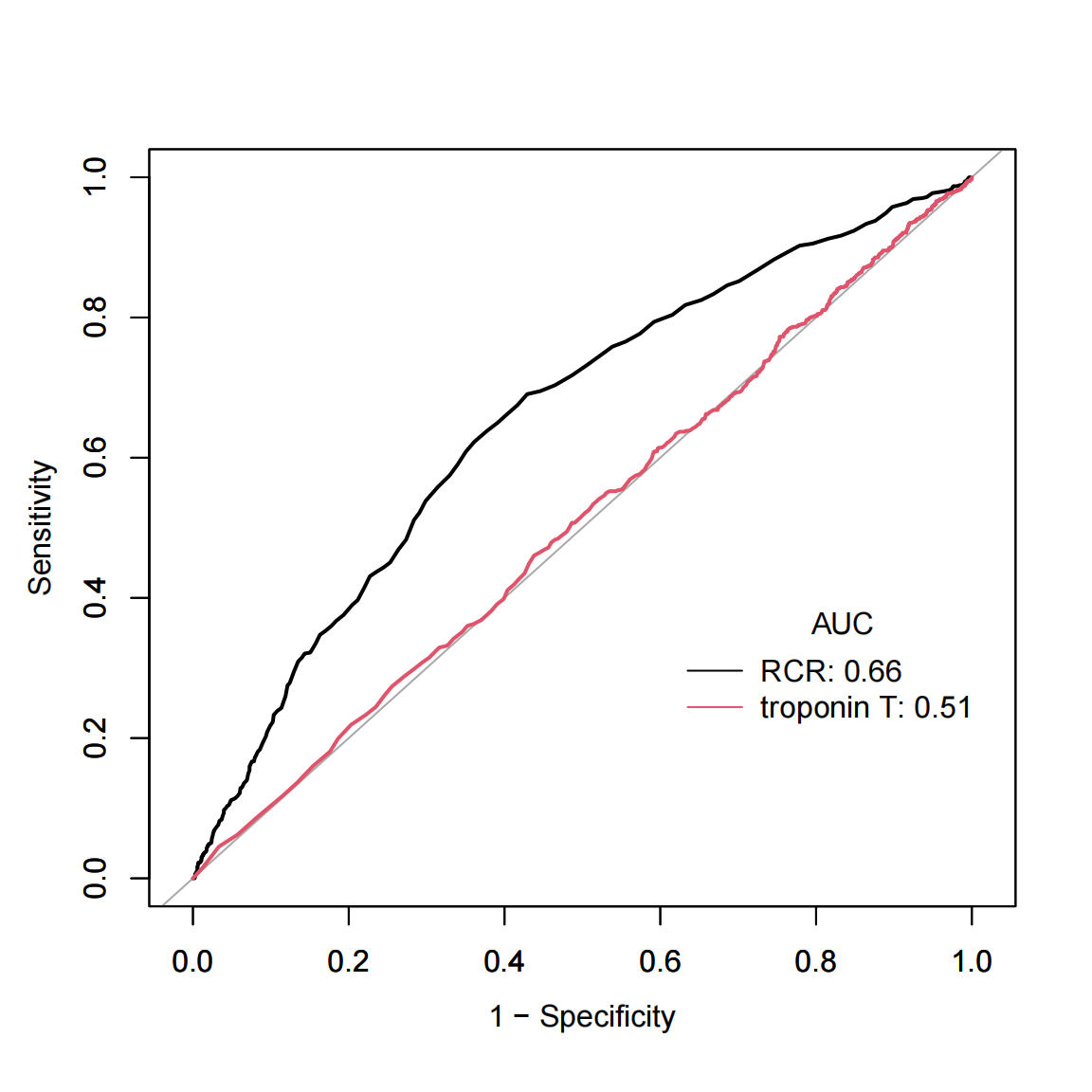

Supplement: Supplementary file 3 [file Image3.tif]

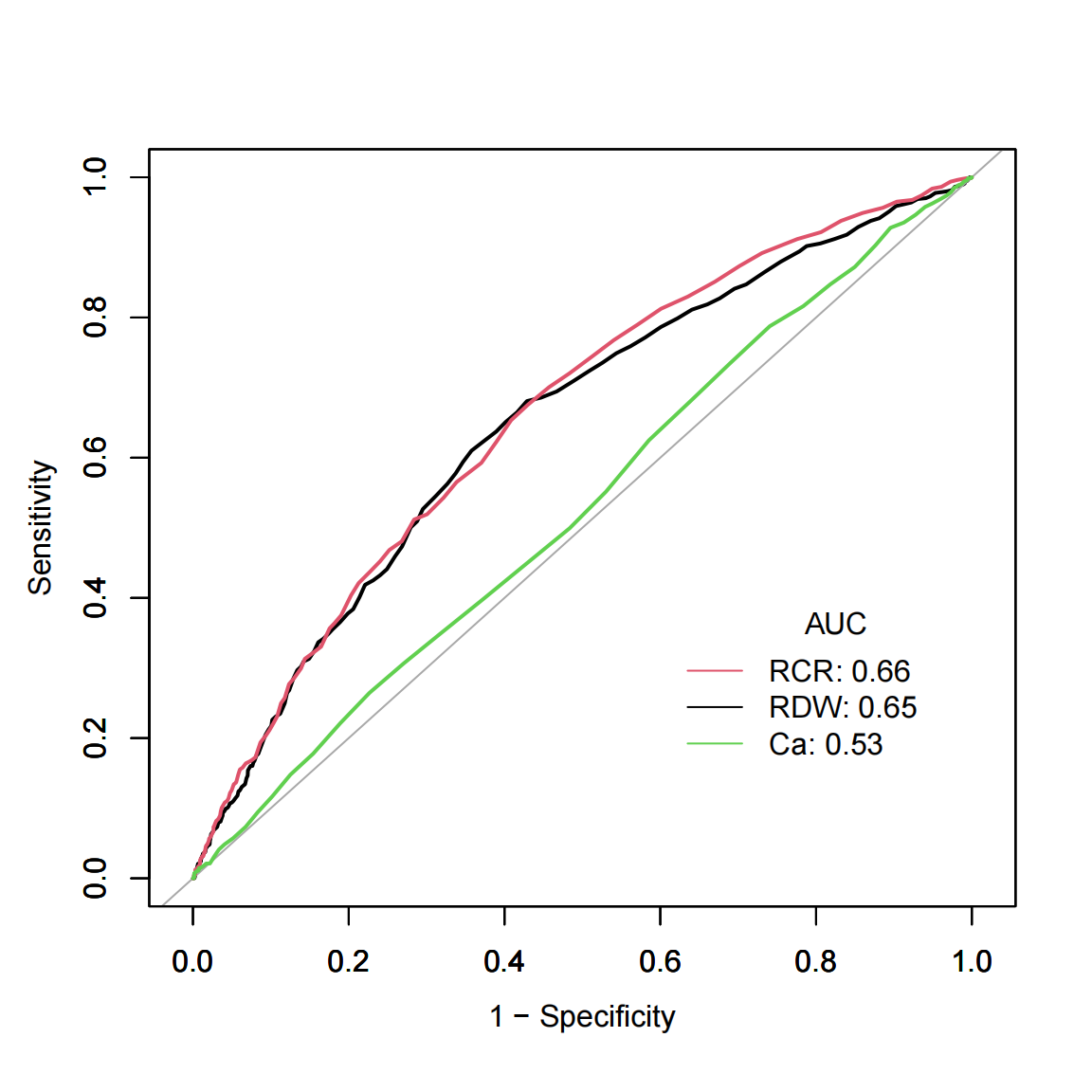

Supplement: Supplementary file 4 [file Image4.tif]
